# The influence mechanism analysis on the farmers’ intention to adopt Internet of Things based on UTAUT-TOE model

**DOI:** 10.1038/s41598-024-65415-4

**Published:** 2024-07-01

**Authors:** Lianying Li, Xin Min, Jinyong Guo, Feng Wu

**Affiliations:** School of Economics and Management & Rural Revitalization Strategy Research Institute, Jiangxi Agricultural University, Nanchang, 330045 China

**Keywords:** Environmental social sciences, Psychology and behaviour

## Abstract

Internet of Things (IoT) technologies are widely recognized as one of the most important infrastructures for economic development and technological innovation. By analyzing the influencing factors of vegetable farmers’ intention to adopt agricultural IoT, it helps to formulate effective IoT promotion policies and accelerate the realization of agricultural modernization. Based on the Unified Theory of Acceptance and Use of Technology (UTAUT) and the Technology-Organization-Environment (TOE) theory, this study constructed for the first time a mechanism model of the influence of vegetable farmers’ intention to adopt IoT, expanding the scope of current research on agricultural IoT and revealing the intrinsic influence mechanism of farmers’ adoption of IoT technologies. In this study, 357 quantitative data were obtained by a questionnaire survey, and structural equation modeling was used to test the direct and indirect effects of each factor on vegetable farmers’ intention to adopt IoT. The results show that almost all variables in TOE have significant direct impacts on the intention, while no variables in UTAUT have significant direct impacts. Among variables in TOE, government support and complexity are the two most important elements influencing the intention. Although the interactions among variables in TOE and UTAUT are also found, the indirect effects of variables are non-significant. Therefore, it is proposed to reduce the complexity of operation and use of IoT technologies; improve rural information infrastructure and compatibility of IoT platforms and devices; and governments should increase subsidies, and incentives to promote the use of IoT in agriculture and agricultural practices.

## Introduction

The “No. 1 central document” of the Central Government of China has, for four consecutive years (from 2020 to 2023), set the goal of ensuring China’s food security and the supply of “vegetable basket” products by promoting the development of smart agriculture and accelerating agricultural modernization. Digital reform and the application of information technology have greatly affected China’s agriculture, rural areas, and farmers, and are leading the agricultural industry to the middle and high end. The Internet of things (IoT) technology has a considerable benefit on minimizing food waste, water waste, carbon emissions, the application of pesticides and chemical fertilizers, enhancing soil management, agricultural productivity, income, and changing traditional human production methods and lifestyle^1^. As a strategic emerging industry, the IoT technology has become a key driver of China’s agricultural modernization, and also an important symbol and trend of modern agricultural development in China^2^.

China is the greatest vegetable producer in the world. However, the sustainability of the vegetable industry is facing many constraints, which necessitate industrial adjustment and production optimization through IoT technologies for further development to occur^3^. IoT technologies are widely recognized as one of the most important infrastructures for economic development and technological innovation. Vegetable farmers are direct customers of IoT technologies, and their actual attitudes towards adoption will affect vegetable industry modernization. An increasing number of scholars has studied these users’ intention and behavior toward IoT technology adoption. The factors influencing the intention of adopting IoT technologies have been classified into four categories according to studies. The first is technical factors. Attributes of the new technology, e.g., farmers’ adoption and acceptance of IoT technologies is influenced by a variety of factors, including technology cost, integration, and usefulness^4^. Takagi, Purnomo, and Kim^5^found that new technologies are more likely to be adopted by farmers if they are compatible and easy to use, and increase agricultural yields and farmers’ income. The second is individual and organizational factors. Studies have found that personal factors, e.g., gender, age, innovativeness, knowledge, and skill characteristics, influence users’ intention to use IoT technologies^6^. Organizational factors refer to top-level support, organizational readiness, farmers’ associations, organization of training activities, and so on. The third category is economic factors. The ability of new technologies to meet performance expectations, reduce financial costs, and achieve perceived benefits will be an important consideration for farmers in deciding whether to adopt them^6^. The final category comprises other external factors, e.g., competitive pressures, government support, environmental changes, and socio-demographic factors^7^.

In terms of research methodology, scholars have empirically investigated the factors of IoT technology adoption mainly from the perspective of theories related to information technology adoption. Most of these studies have been conducted using the Technology Acceptance Model (TAM model)^8^, the Unified Theory of Acceptance and Use of Technology (UTAUT)^9^, the Technology-Organization-Environment (TOE) model^10^, and UTAUT + TOE coupled model^11^. Other empirical methods have also been used. For example, Shi et al. applied Logistic-ISM to 52 agribusinesses and found a hierarchy of factors influencing their adoption intention^12^. Structural equation modeling was used to study the impact of perceived usefulness, perceived enjoyment, and perceived privacy risk on IoT adoption among users^13^.

To summarize, the existing studies exploring the influence mechanism of IoT technology adoption intention have laid a good foundation for this study, but there is still room for the following expansion, and this study may be innovative in the following areas:

1. Concerning the adoption of IoT technologies, research in developed countries started early and has yielded many results^5,7,14,15^. Extant research on IoT technology adoption in China has mostly focused on the theoretical level. Less empirical research has been done on IoT adoption intentions, particularly in agriculture.
2. Existing models do not reflect the intrinsic interaction among various factors and the mechanisms that further influence the intention to adopt IoT technologies. Most existing studies on technology and information adoption are based on a single-model framework^10,12,16^, e.g., UTAUT, TOE, and there are few studies coupling the two theories to complement each other.

So, what are the factors influencing vegetable farmers’ intention to adopt IoT? Is there an intrinsic structure and interaction between these factors? How do these factors contribute to vegetable farmers’ intention to adopt IoT? What are the key factors determining their intention? What are the influencing mechanisms? These issues are urgent problems in the formation process of vegetable farmers’ intention to adopt IoT in China. In this regard, based on UTAUT and TOE theories, this study constructs a theoretical model, explores the influencing factors of vegetable farmers’ intention to adopt IoT, reveals the internal structure and interaction between these factors, and how these factors act on vegetable farmers’ intention to adopt IoT. This study can help to supplement and improve the research on the adoption intention of agricultural IoT, enhance the application of IoT in agriculture, realize the empowerment of the vegetable industry by IoT technologies, and promote the process of agricultural modernization.

## Literature review

### Overview of UTAUT and TOE models

UTAUT was proposed by Venkatesh et al.^28^ and is based on the Technology Acceptance Model proposed by Davis. It is an integration of findings related to the Theory of Rational Behavior (TRA), Theory of Planned Behavior (TPB), Technology Acceptance Model (TAM), Motivation Model (MM), TAM-TPB Composite Model (C-TAM-TPB), Model of PC Utilization (MPCU), Innovation Diffusion Theory (IDT), and Social Cognitive Theory (SCT). Venkatesh proposed a second-generation integrated model of technology acceptance and use that extends UTAUT to the individual consumer domain, by enhancing the initial generation of UTAUT with hedonic drive, price value, and habit, and removing voluntariness as a moderating variable. UTAUT is a well-established theoretical model that explains 77% of the variance in behavioral intention to use a technology and 52% of the variance in technology use in studies of individual use of technology, and is predictive in a variety of domains and across technology adoptions^17^, e.g., IoT technologies^18^, IoT + food traceability technologies^19^, self-driving car technology^20^, and e-commerce^21^.

The TOE framework was developed by Tornatzky and Fleischer^23^ on the basis of the critical theory of diffusion of innovation to study and synthesize various factors affecting firms’ adoption of innovative technologies^22^. Compared with Innovation Diffusion Theory (IDT), which only considers technological and organizational factors of technology adoption, the TOE framework is regarded as being more efficient and adaptable in terms of industry application and organizational size^23^. The TOE framework is widely used in studies of innovation adoption and its influencing factors in organizations that are involved in open data-sharing^24^, enterprise resource planning^25^, artificial intelligence embedded technology^26^, etc. In the TOE framework, the factors influencing IT adoption are integrated into three dimensions: technology, organization and environment. The technological dimension mainly includes the basic technological conditions that are available (e.g., technological infrastructure, technological capabilities, and so on) and the characteristics of the technology use (e.g., relative advantages of the technology, compatibility, cost, and complexity). The organizational dimension encompasses the corporate structure, innovation capabilities, knowledge capabilities, operational capabilities, strategic use of technology, trust, technological resources, top management support, quality of human capital, and expertise and infrastructure^27^. The organization’s specific environment is represented by the environmental dimension, including its industry, competitors, suppliers and relationships with government entities^22^. This theory is suitable for explaining and analyzing influencing factors of technology adoption at the firm level.

### Theoretical model construction

**Figure 1:**
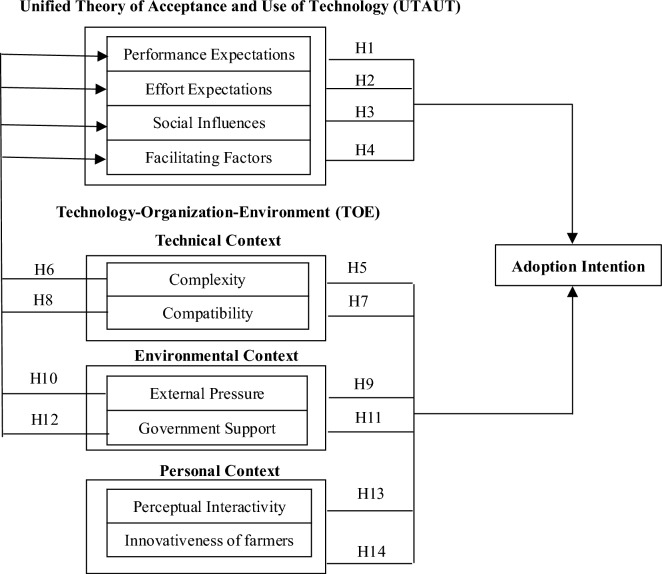
Research model of intention to adopt IoT technologies among vegetable farmers.

However, existing studies of IoT technology adoption intention lack a unified recognized authoritative theoretical model. Although the UTAUT model is the core part of integrating several theories of technology adoption and also has a higher level of reliability and validity, but is lacking research on the users of individual information system decision-making behavior of “organizational people” (i.e., those who are in an organizational context and are mandatorily subject to organizational rules). Furthermore, UTAUT has its own research hypothesis limitations. Therefore, it is necessary to find other variables that are more in line with the characteristics of IoT technologies. This requires modifying and extending the model somewhat to give it more explanatory power. In contrast, the TOE model has the advantages of being systematic and thorough in analyzing the key factors of IoT adoption from three perspectives: technology, environment, and organization, in a comprehensive manner. IoT technology is still in the demonstration application stage in China, and itself has characteristics such as difficulty and complexity. Therefore, in order to make the constructed model most appropriate for the study of the actual situation of IoT technology adoption intention of vegetable farmers in China, the purpose of this study is to merge the UTAUT and TOE models while taking into account the characteristics of vegetable farmers and IoT technologies. This study specifically picks the key variables in both the UTAUT model (performance expectations, effort expectations, social influence, and facilitating factors) and the TOE model (technical context, environmental context, and personal context) to construct the model for vegetable farmers’ intention to adopt IoT technologies (See Fig. 1).

## Research hypotheses

### Individual context

#### Performance expectations, effort expectations, social influences, and facilitating factors

Performance expectations are the degree to which people believe that using IoT technologies would help them perform better at work^28^. Farmers will adopt IoT technologies when they see clear value, benefits, and return on investment from using them^29^. In other words, performance expectations influence technology adoption positively. As a new technology in vegetable cultivation, farmers’ expectations of its performance may impact its acceptability and utilization. As a result, the following research hypothesis is proposed in this paper:

H1: Performance expectations will have a significant positive impact on the intention to adopt IoT technologies among vegetable farmers.

Effort expectations depend on how easily they can use IoT technologies, which directly influences their decision to use it^28^. IoT technologies reduce costs for users and bring great changes to production and sales. However, specific equipment must be installed to use IoT technologies; therefore, the easier IoT technology is to operate in practice, the greater its direct positive impact on users^30^. As a result, the following research hypothesis is proposed in this paper:

H2: Effort expectations will have a significant positive impact on the intention to adopt IoT technologies among vegetable farmers.

Social influence is the extent of influence that people perceive coming from their immediate social and political environments, including from other individuals, the government, the media, and their family and friends^28^. Previous studies have confirmed the significance of social influence on adoption intention^18^. As a result, the following research hypothesis is proposed in this paper:

H3: Social influence will have a significant positive impact on the intention to adopt IoT technologies among vegetable farmers.

Facilitating factors are the extent to which people think the current system and technological infrastructure can support system use. Specifically, they are extent to which people believe that the convenience and different technical support conditions required for the successful use of IoT technologies. Facilitating factors, as one of the core variables, directly determine behavioral intention to use technology^31^. The ease of access and availability of technical support considerably improved farmers’ intention to adopt IoT technologies in studies of its adoption by fruit farmers^30^. Correspondingly, this paper proposes:

H4: Facilitating factors will have a significant positive impact on the intention to adopt IoT technologies among vegetable farmers.

### Technology context

#### Complexity and compatibility

Complexity means the ease of adoption, comprehension, and use of the technology^32^. The more complex the IoT technologies are, the more challenging vegetable farmers perceive obtaining better work performance to be, the more difficult the technology is to use, the lower the existing system support, and the lower the intention to adopt. Correspondingly, this paper proposes:

H5: Complexity will have a significant, negative, and direct impact on the intention to adopt IoT technologies among vegetable farmers.

Similarly, complexity may reduce individual context and indirectly impact their adoption. If an organization’s systems are complex, users will have trouble using new technologies and thus have difficulty in recognizing the value of the new system^33^. Therefore, complexity may reduce performance expectations. Perceived usefulness and perceived ease of use decrease as technology complexity increases^26^. It has also been argued that complexity has a negative impact on other individual factors, such as social influence and facilitating factors^34^. Therefore, we propose:

H6: Complexity will have a significant negative impact on individual context of IoT technologies’ adoption among vegetable farmers.

Compatibility refers to the degree of adaptation of IoT technologies and organizational elements^32^. In this study, it mainly includes the objectives to be achieved by the adoption of IoT technologies, and the degree of matching the existing software and hardware facilities, information systems, and business processes. Existing studies show that the higher the degree of matching, the easier it is to adopt the technology^5^. Therefore, this paper proposes:

H7: Compatibility will have a significant, positive, and direct impact on the intention to adopt IoT technologies among vegetable farmers.

Besides the direct impact, compatibility may affect individual context and have an indirect impact on the adoption. Compatibility will increase the user’s perceived benefits and convince him/her that using the IoT will improve his/her work/life performance^26^. Compatibility has also been shown to significantly influence effort expectations^35^, social influence^36^ and facilitating factors^37^. Therefore, we propose:

H8: Compatibility will have a significant positive impact on individual context of IoT technologies’ adoption among vegetable farmers.

### Environmental context

#### External pressure and government support

External pressure refers to the pressure from peers, the social environment, and partners. External pressure is frequently employed to investigate the influence of external factors on technology adoption. It has been well documented that the more external pressure users feel, the weaker the rate of technology adoption^38^. Therefore, we propose:

H9: External pressure will have a significant, negative, and direct impact on the intention to adopt IoT technologies among vegetable farmers.

In addition, studies confirm that external pressure influences individual context positively^15^. For example, Rodríguez-Espíndola et al. found that regulatory guidance can influence users’ perceptions and ultimately enhance intentions to use emerging technologies, while Xu confirmed that environmental context (competitive pressure, trend pressure, and pressure to enforce norms) have a positive impact on performance expectations, effort expectations, social influences and facilitating factors^15,34^. Based on the above research results, this paper proposes:

H10: External pressure will have a significant positive impact on individual context of IoT technologies’ adoption among vegetable farmers.

Government support refers to the human, material, and financial resources, as well as infrastructure, provided by national and local governments. In China, the application of IoT technologies in agriculture is still in its early stages, and the input cost of using IoT technologies is still relatively high; therefore, government support will be a powerful vehicle for promoting IoT technology application^24^. Thus, we propose:

H11: Government support will have a significant positive impact on the intention to adopt IoT technologies among vegetable farmers.

In the use of technology in corporate innovation ecosystems, government policy, legal, and other forms of assistance have a considerable positive impact on employee-level(perceived usefulness, perceived ease of use, etc.)^30^. Xu argued that government support assistance benefits both the social influence and facilitating factors^34^. Thus, we propose:

H12: Government support will have a significant positive impact on individual context of IoT technologies’ adoption among vegetable farmers.

### Personal context

#### Perceptual interactivity and innovativeness of farmers

Perceptual interactivity means the degree to which vegetable producers may participate in IoT information communication and exert influence or react to IoT technologies. Perceptual interactivity can improve users’ intention to adopt by improving the quality of information communication in IoT and enhancing users’ positive experience of the information communication process^39^. Thus, this paper proposes the following research hypothesis:

H13: Perceptual interactivity will have a significant positive impact on the intention to adopt IoT technologies among vegetable farmers.

In the general TOE framework, major corporate decisions are implemented by top executives^38^. Vegetable farmers are the equivalent of executives in a business or organization, who hold the power of decision over the adoption of IoT technologies. In general, highly innovative individuals are constantly seeking out new technologies or thoughts, have the capacity to deal with uncertainty, and are more likely to embrace technology^32^. In addition, empirical evidence also confirms that individual innovativeness has an important effect on adoption of IoT technologies^7^. Thus, this paper proposes the following research hypothesis:

H14: Innovativeness of vegetable farmers will have a significant positive impact on the intention to adopt IoT technologies among vegetable farmers.

## Materials and methods

### Data collection

Data for this study were collected from 20 counties, cities, and districts in Jiangxi Province, China, with a high concentration of vegetable farmers during the period June 2022-June 2023. Stratified random sampling can improve the precision of the estimation of the sample indicators to the overall indicators, so that the sample is more evenly distributed in the overall, the representativeness of the stratified random sampling sample is better, and the sampling error is smaller, therefore, this study adopts stratified random sampling, from which three sample townships are selected, and in each sample township, 10 households are randomly selected. In total, 600 questionnaires were distributed, 406 were collected, 357 were valid, and the effective recovery rate was 64.9%. Men accounted for 90.2% (322), women 9.8% (35); 30 to 59 years old accounted for the majority (88.8%), below 30 years old accounted for 4.8%, and above 60 years old accounted for 6.4%; 212 people with junior high school education or below accounted for 59.4%; married accounted for 95.2%, and unmarried accounted for 4.8%; the number of vegetable cultivation 10 to 49 acres accounted for 61.6%, 50 to 99 acres accounted for 12.9%, and more than 100 acres accounted for 25.5%.

### Questionnaire design

This study’s formal questionnaire is divided into three sections. The first section is to gather data on the regional distribution of vegetable farmers. The second section includes questions about demographic, family, production, and managerial aspects, and the intention to adopt IoT technologies. The third section included scales linked to the elements that influence adoption intention. This study adopted the proven scale used by other scholars (see Table 1) and adapted the scale to the actual situation of vegetable IoT in China, including 11 measurement elements. The measurement of the research variables followed the practice of other scholars and the corresponding values were obtained using a five-point Likert scale, with the numbers “1, 2, 3, 4, and 5” meaning “strongly disagree, disagree, average, agree, and strongly agree”, respectively. (For a complete description of variables and supplementary statements, see Table 1).

**Table 1:** Variables, associated statements, and adoption intention.

Variable	Statements	Average	SE	Document source
Performance Expectations				
PE1	Using IoT technologies can benefit me	3.58	0.048	Venkatesh et al. 2003^28^
PE2	The use of IoT technologies can improve the efficiency of vegetable farming and save time	3.74	0.044	
PE3	Using IoT technologies can reduce labor costs	3.76	0.046	
Effort Expectations				
EE1	I believe that learning how to use the Internet of Things is easy	2.65	0.062	Venkatesh et al. 2003^28^
EE2	I don’t think it’s complicated to use the IoT to operate	2.58	0.062	
EE3	Using IoT technologies to accomplish task-specific operations is easy for me	2.55	0.066	
EE4	It was easy for me to learn to become proficient in the use of IoT	2.48	0.065	
Social Influence				
SI1	Other vegetable-farmers are using (or about to use) IoT technology	2.38	0.063	Venkatesh et al. 2003^28^
SI2	I have been encouraged to use IoT by the government and the mass media	2.71	0.061	
SI3	Friends and family encourage me to use IoT technologies	2.54	0.061	
Facilitating Factors				
FC1	I have a relatively complete information technology infrastructure	2.08	0.064	Venkatesh et al. 2003^28^
FC2	I have specialized IT personnel or teams	1.93	0.062	
FC3	I have relevant experience in implementing IT or information system projects	1.98	0.064	
*Complexity*				
CO1	The IoT system is complicated to operate in practice	3.54	0.051	Oliveira et al. 2014^40^
CO2	The IoT system is not easy to use	3.00	0.059	
CO3	Extensive experience is required to use the IoT system	3.43	0.054	
Compatibility				
CE1	I already have equipment and software that can handle the needs of IoT technologies	2.41	0.070	Oliveira et al. 2014^40^
CE2	IoT technology can integrate with my other information systems (e.g., ERP, MIS, etc.)	2.42	0.067	
CE3	IoT technology integrates well with my business processes	2.68	0.063	
External pressure				
EP1	External competitive pressures necessitate the adoption of IoT technologies	4.02	0.052	Oliveira et al. 2014^40^
EP2	Social factors (culture, customs, etc.) influence the adoption of IoT technologies	3.95	0.044	
EP3	Partners demand the adoption of IoT technologies	3.87	0.045	
*Government support*				
GS1	The government provides funding for the development of IoT technologies	3.04	0.060	Yee-Loong & Ooi, 2008^41^
GS2	The government introduces relevant policies to strongly support the development of IoT technologies	3.22	0.057	
GS3	Government provides talent support for the development of IoT technologies	2.93	0.059	
Perceptual interactivity				
PI1	I can obtain the necessary information through the Internet of Things	3.27	0.060	Su 2014^42^
PI2	I can collect the IoT feedback you require	3.40	0.058	
PI3	I can interact with other IoT items (people, objects, systems, and so forth)	3.35	0.058	
*Innovativeness of farmers*				
LF1	I like to experiment with everything	3.08	0.058	Shi et al. 2022^30^; Rogers 2002^32^
LF2	I’m always the first of my peers to try a new technology	2.88	0.064	
LF3	I don’t hesitate to try a new technology	3.04	0.061	
Adopt intention				
AI1	I would use or start using applications of IoT technologies	2.79	0.065	Venkatesh et al. 2003^28^
AI2	I would like to learn how to use IoT applications	3.13	0.063	
AI3	While I use IoT applications, I would recommend them to others	3.12	0.063	

### Ethics declarations

All methods were carried out in accordance with relevant guidelines and regulations. All experimental protocols were approved by the Jiangxi Agricultural University. Informed consent was obtained from all subjects and/or their legal guardian(s).

## Empirical analysis

### Measurement model testing

The internal consistency coefficient (Cronbach’s α coefficient) reliability method was used to test the reliability or stability of the measurement results. Table 2 shows that the Cronbach’s alpha values for all latent variables far exceed 0.7, with the vast majority between 0.8 and 0.9, indicating that the reliability is high.

**Table 2:** Results of reliability and validity analysis of each variable.

Latent variable	Number of items	Cronbach’s Alpha value	KMO
Performance expectations	3	0.859	0.711
Effort expectations	4	0.924	0.833
Social influence	3	0.806	0.695
Facilitating factors	3	0.933	0.764
Complexity	3	0.728	0.663
Compatibility	3	0.858	0.668
External pressure	3	0.830	0.683
Government support	3	0.768	0.691
Perceptual interactivity	3	0.884	0.727
Innovativeness of farmers	3	0.883	0.738
Adopt intention	3	0.832	0.682

The validity of the scale covers both content validity and construct validity. The scale selected for this study is a mature scale that has been used widely. The construct validity of the scale was evaluated for exploratory factor analysis. To determine whether the inter-items were adequate for factor analysis, the Kaiser–Meyer–Olkin (KMO) measure of sample adequacy value and Bartlett’s test might be utilized. When the KMO value was above 0.60, which implied Bartlett’s test yielded significance, factor analysis could be conducted; when the KMO value was less than 0.50, it indicated that factor analysis was not suitable. Table 2 shows that the scales’ KMO values are more than 0.65 and Bartlett’s test indicated significance. Therefore, the scales in this paper are suitable for factor analysis.

CFA was executed on all items and their corresponding latent variables using AMOS 17.0 to test the hypothesized factor structure and data fit. The measurement model fit the data well. Therefore, the relationships between variables could be further analyzed.

### Structural equation model testing

The technical fit parameters of the SEM were as follows (Table 3): χ^2^/df = 2.980, RMSEA = 0.075, NFI = 0.824, CFI = 0.875, and GFI = 0.787. Table 3 shows that the absolute, relative, and parsimonious fit indexes were near to the adaption standard, indicating that the SEM output had a good overall fit and could be utilized to evaluate the proposed theoretical hypotheses.

**Table 3:** Structural equation model fit indices.

	Fitting index	Model estimation	Explanation
Absolute fit index	χ^2^	χ^2^ = 1493.224 (*df* = 501)	Significant
	GFI	0.787	≈0.8, good
	AGFI	0.747	≈0.8, good
	RMSEA	0.075	< 0.08, very good
Relative fit index	CFI	0.875	≈0.9, very good
	IFI	0.876	≈0.9, very good
	NFI	0.824	> 0.8, good
	RFI	0.803	> 0.8, good
	TL	0.860	≈0.9, very good
Parsimonious fit index	AIC (Theoretical model)	1681.224	AIC (Theoretical model) < AIC (Standalone mode)
	AIC (Saturation mode)	1190.000	
	AIC (Standalone mode)	8542.947	
	NC	2.980	1 < NC < 3, good fit
	PNFI (Concise standard fitting index)	0.736	> 0.5, very good
	PGFI (Concise comparison and fitting index)	0.781	> 0.5, very good

### Research hypothesis testing and discussion

The parameter estimation result of the SEM model is provided in Table 4 and the results from testing the hypotheses in Section. 2.3 are presented in Table 5. Some interesting findings are stated below. First, Existing studies generally agree that the UTAUT variable affects adoption intentions^6,18,30,31^. However, in this study, the performance expectations, effort expectations, social influence, and facilitating factors in UTAUT did not have a significant positive impact on the intention to adopt IoT technologies. Accordingly, the research hypotheses H1, H2, H3, and H4 are not supported (Table 5). These results are consistent with the reality observed regarding the development of agricultural IoT technologies among Jiangxi farmers. Jiangxi agriculture IoT technologies are still in their early stages of development^43^. Specifically, most vegetable farmers (59.4%) have just a junior high school education or less; they are mostly elderly (75.9%) (over 40 years old), and their knowledge of agricultural IoT technologies is limited. They do not fully comprehend the characteristics of agricultural IoT technologies; as a result, they do not have a strong perception of IoT technologies as enhancing work performance or being easy to use. Thus, performance expectations and effort expectations had no substantial positive impact on adoption intention (H1 and H2). The adoption of IoT technologies to grow vegetables is dominated by agricultural enterprises. It is difficult for ordinary vegetable farmers to learn and use agricultural IoT technologies^44^. Their knowledge of IoT technologies is only superficial. Also, it is challenging for vegetable farmers to directly reference the effects of adoption of IoT technologies by other vegetable farmers. Thus, social influence and facilitating factors do not have a significant impact on the intention to adopt IoT technologies (H3 and H4).

**Table 4:** Path coefficients of the theoretical model.

Regression path	Coefficients	S.E	C.R
Individual Context			
performance expectations → adoption intention	− 0.028	0.067	− 0.418
effort expectations → adoption intention	− 0.01	0.055	− 0.172
social influence → adoption intention	− 0.084	0.076	− 1.11
facilitating factors → adoption intention	0.003	0.051	0.056
Technical Context			
complexity → adoption intention	− 0.496***	0.104	− 4.766
complexity → performance expectations	− 0.530***	0.082	− 6.469
complexity → effort expectations	− 0.638***	0.1	− 6.359
complexity → social influence	− 0.338***	0.084	− 4.034
complexity → facilitating factors	− 0.421***	0.093	− 4.513
compatibility → adoption intention	0.144**	0.061	2.348
compatibility → performance expectations	0.006	0.047	0.127
compatibility → effort expectations	0.341***	0.062	5.514
compatibility → social influence	0.324***	0.057	5.648
compatibility → facilitating factors	0.703***	0.072	9.767
Environmental Context			
external pressure → adoption intention	0.021	0.066	0.311
external pressure → performance expectations	0.203**	0.069	2.947
external pressure → effort expectations	0.047	0.083	0.56
external pressure → social influence	− 0.168**	0.077	− 2.194
external pressure → facilitating factors	− 0.208**	0.084	− 2.469
government support → adoption intention	0.592***	0.092	6.418
government support → performance expectations	0.231***	0.065	3.541
government support → effort expectations	0.355***	0.085	4.192
government support → social influence	0.501***	0.087	5.772
government support → facilitating factors	0.245**	0.083	2.954
Personal Context			
perceptual interactivity → adoption intention	0.171***	0.043	4.016
innovativeness of farmers → adoption intention	0.149**	0.047	3.179

Notes: * Significant at the 10% level; ** significant at the 5% level; and *** significant at the 1% level.

**Table 5:** Hypothesis tests of the theoretical model.

Hypothesis	Description	Test results
Individual Context		
H1	performance expectations → adoption intention	Not supported
H2	effort expectations → adoption intention	Not supported
H3	social influence → adoption intention	Not supported
H4	facilitating factors → adoption intention	Not supported
Technical Context		
H5	complexity → adoption intention	Supported
H6	complexity → individual context	Supported
H7	compatibility → adoption intention	Supported
H8	compatibility → individual context	Supported
Environmental Context		
H9	external pressure → adoption intention	Not supported
H10	external pressure → individual context	Not supported
H11	government support → adoption intention	Supported
H12	government support → individual context	Supported
Personal Context		
H13	perceptual interactivity → adoption intention	Supported
H14	innovativeness of farmers → adoption intention	Supported

Second, all variables in TOE, with the exception of external pressure, have significant impacts on the intention. Among them, complexity was found to have a significant negative impact (H5). Complex technology will take more time for farmers to learn, which will impose costs and risk on them, thus hindering the adoption of IoT, this result further confirms that technological complexity is a hindrance to the adoption of IoT by vegetable farmers^45^. Compatibility had a significant positive impact on the intention (H7). Compatibility with existing technologies helps farmers understand IoT and also increases their closeness to them. Also, farmers are more able to anticipate the benefits of adopting IoT through comparison with existing technologies^5^. External pressure does not have a significant impact on the adoption of IoT (H9), this is contrary to the findings of Adu-Gyamfi^46^. The reason may be that, in Jiangxi, vegetable cultivation is still dominated by traditional cultivation methods, and transactions are usually made in the provincial market. There is no excessive demand and pressure from vegetable cultivation counterparts and partners for the adoption of refined IoT cultivation. Government support has a significant positive impact on the adoption (H11). The government is an essential participant in the promotion of IoT technologies^30^. By providing financial subsidies and policy support, the government can enhance the recognition of IoT technologies by farmers and increase their intention to use it. Perceptual interactivity significantly affects adoption intention (H13), because vegetable farmers can perceive their active participation in the process of using IoT and receive timely feedback, which can help vegetable farmers better understand and grasp the features and functions of IoT, our finding further confirming the importance of perceptual interactivity on adoption intentions^39^. Innovativeness of farmers has a significant positive impact on adoption intention (H14). IoT is a new technology in agriculture. When vegetable farmers have the willingness and ability to accept new technology, their adoption intention will also increase^7^.

Third, given that none of the individual context have a direct effect on the intention to adopt, the indirect effects of all TOE variables on the adoption intention, through their influence on individual context, are non-significant. However, it is still meaningful to observe those significant effects of TOE variables on individual context because the effect of individual context on adoption intention changes with the development of agriculture, and the indirect effect might be significant in the future. This is a test of whether four individual context are jointly affected by each TOE variable, and the test results are presented in Table 5. Among them, complexity has a negative impact on individual context. This is consistent with the reality that the more complicated the technology, the more difficult it is for vegetable farmers to master it, and therefore has a significant negative effect on individual context (H6). Compatibility has a positive impact on individual context, and refers to the extent of matching with the existing technology: the higher the compatibility, the more easily vegetable farmers can accept and use the IoT (H8). External pressure does not have a significant positive impact on individual context(H10), which is contrary to the findings of Rodríguez-Espíndola^15^. The reason may be that, vegetable farmers are still engaged in family-based production and operation, and the vast majority of vegetable farmers sell vegetables to consumers in the local market. Government support has a positive impact on individual context. The government plays the role of regulating and guiding the production activities of vegetable farmers, and can enhance the awareness of the IoT among vegetable farmers and thus significantly impact individual context (H12).

Last, by comparing the path coefficients, we found that government support and complexity are the two most important elements influencing vegetable farmers’ intention to utilize IoT technologies. Government support can provide vegetable farmers with new IoT knowledge, information, and financial subsidies, which can help vegetable farmers better accept and master IoT technologies, and increase their awareness of IoT technologies. Complexity has a significant negative impact on this intention, with a path coefficient of − 0.496, reaching a significant level of 0.001. Because IoT is an emerging technology, its adoption is challenging for vegetable farmers with limited cognition, and the use of some devices is not only beyond their cognitive scope, but also takes time to learn to use.

## Implications

### Theoretical implications

This study investigated the intention of vegetable farmers to adopt IoT in China and used SEM to validate the influence mechanism of vegetable farmers’ intention to adopt IoT, expanding the UTAUT model in the following three aspects:

First, based on UTAUT and TOE, this study constructed a model of the influence mechanism of vegetable farmers’ IoT adoption intention for the first time. Previous studies have widely applied UTAUT or TOE to the study of technology adoption intention^18–21^, but our study goes a step further by applying them to the field of agricultural technology adoption. In related studies in the field of agricultural technology, scholars have mainly investigated the influence mechanisms of technology adoption such as fertilizer and pesticide reduction technology, water-saving irrigation technology, and soil testing and formulation technology, and the study of vegetable farmers’ intention to adopt under the context of the IoT in agriculture has not yet been fully explored. This study provides a more complete research framework for the study of vegetable farmers’ intention to adopt IoT and analyzes the influence mechanism of vegetable farmers’ intention to adopt IoT accordingly. An avenue of future research is to follow the latest development of UTAUT and TOE to provide a more comprehensive and enriched research framework for the study of adoption intention.

Second, this study is the first to examine both the direct and indirect effects of internal and external factors on vegetable farmers’ intention to adopt IoT. Previous studies have elucidated how internal and external factors directly affect vegetable farmers’ intention to generate IoT adoption^24^, but this study extends the literature by focusing on the relationship between UTAUT and TOE variables. The results of the study show that UTAUT variables do not have a direct impact on vegetable farmers’ adoption of IoT, however, TOE variables almost always have a significant impact on vegetable farmers’ adoption of IoT. We tried to consider the interactions between the variables in TOE and UTAUT and found that the interaction between external pressures and the UTAUT variables was not significant, probably because vegetable farmers in different regions have different resources, environments, and market conditions, as well as differences in vegetable farmers’ cultural backgrounds and values. Although the indirect effect on vegetable farmers’ intention to adopt IoT is not significant, this study provides scholars with perspectives for further exploration. We propose that future scholars need to consider the interactions between the factors influencing vegetable farmers’ intention to adopt IoT in greater depth, taking into account geographical and cultural differences, in order to more fully understand the mechanisms influencing vegetable farmers’ intention to adopt IoT.

Third, this study expands the understanding of the applicability of UTAUT and TOE, further improving the explanatory power and breadth of application of the theory. Previous studies have confirmed the appropriateness of the application of UTAUT in the information technology field, suggesting that UTAUT variables promote technology adoption^18,19^, but some scholars have found that UTAUT focuses too much on individual factors, and combining UTAUT with other theories can help to improve the explanatory power of the model^17^. Although this study coupled UTAUT and TOE to establish a mechanism model, it was still found that the influence of UTAUT variables on vegetable farmers’ intention to adopt IoT was not significant. The reason for this may be because in addition to the four moderating variables (gender, age, experience, and voluntariness) proposed by the UTAUT model, factors such as organizational model should be also selected to moderate the intention to adopt according to the actual situation of technology adoption. Therefore, future scholars can further consider the special characteristics of vegetable farmers and the characteristics of agricultural IoT in their research, and include gender, age, farming experience, and organizational mode as moderating variables in the research model. On the other hand, although this paper covers as many of the more important variables as possible in the course of the research, there may be technological context, environmental context, and personal context selected from the TOE framework that have been omitted. In the future, scholars can select as many variables as possible among the technological, environmental, and personal context for exploration.

### Practical implications

First, to promote adoption intention, the complexity of operating and using IoT technologies should be reduced through, e.g., the layout and construction of industry-academia-research practice bases, the development of a socialized agricultural science and technology service system, in-depth implementation of science and technology commissioner system, encouraging scientific researchers in research and production to communicate with the public in order to understand barriers or difficulties in the use of IoT technologies, and thus simplify their technical operation and use. In regard of this, the government should encourage and support universities, especially agricultural colleges, to strengthen ties with vegetable farmers, build research and practice bases in rural areas, and bring the most advanced theories and technologies to local agriculture and rural areas while conducting teaching and research.

Second, the government should improve rural information infrastructure and the compatibility of IoT platforms and devices, e.g., the existing software and hardware facilities, information systems and business processes in rural areas, promote internet availability in rural areas, and conduct a comprehensive compatibility test for browsers that need to be used for IoT technologies. At the same time, provincial, municipal, and county governments should survey equipment and operating systems used by vegetable farmers, recommend the most suitable equipment and operating systems for them, and provide them with clear and easy-to-understand video operating instructions and technical documents.

Third, government support should be crucial in promoting adoption intentions. Subsidies and preferential policies can be increased by the government, e.g., grant credit loans for vegetable farmers who are willing to adopt IoT-related technologies in vegetable growing. The government can also strengthen training on IoT technology for vegetable farmers, including through the provision of seminars and training courses, to enable vegetable farmers to exchange experiences, share solutions, and learn how to select, deploy, and manage IoT technologies; and improve the ability of vegetable farmers to use IoT technologies. Such training would increase the awareness and understanding of IoT technologies among farmers, and should be complemented with incentives for farmers to increase their intention to adopt IoT technologies. Government departments can also set up IoT vegetable display areas at training sessions or allow farmers to customize their IoT devices according to their needs and preferences to experience more convenience and intelligence. For example, demonstrations or visits to fields or greenhouses where IoT has been successfully applied can be organized so as to allow farmers to see, first-hand, how IoT operates and study its effects in order to enable them to have a more intuitive experience of the value and potential of IoT technologies.

### Research limitations and suggestions for future research

Although this study has some theoretical contributions to the development of UTAUT and TOE theories as well as guiding the application of IoT in agriculture to promote the process of agricultural modernization, the following shortcomings still exist that need to be improved in future research: due to the limitation of the difficulty of questionnaire collection, this study only investigated vegetable farmers in Jiangxi Province, and the sample size collected is limited, and in the future, under the further popularization of agricultural Internet of Things technologies, we can expand the sample size again, and conduct a wider survey based on the whole country. Moreover, there are still fewer measurement studies in China’s academic circles on the variables of IoT adoption intention of vegetable farmers. Chinese academics have conducted fewer measurement studies on the variable of intention to adopt IoT among vegetable farmers, and the scale used in this study mainly adopts the more mature measurement scale proposed by foreign scholars, which has its limitations in terms of applicability in China because the content of the original scale was developed to reflect the actual situation in foreign countries. Although this paper has covered as many important variables as possible in the research process, there may still be some variables that have not been taken into account and need to be improved in future research.

### Supplementary Information

Supplementary Information.

## Data Availability

The raw data and analysis files used to support the findings of this study are available from the corresponding author upon request.
