# Supplementary material for: The influence mechanism analysis on the farmers’ intention to adopt Internet of Things based on UTAUT-TOE model
Source: Sci Rep. 2024 Jul 1;14:15016. doi: 10.1038/s41598-024-65415-4 (PMC11217386; doi:10.1038/s41598-024-65415-4)
Supplement: Supplementary file 1 — Supplementary Information. [file 41598_2024_65415_MOESM1_ESM.docx]

1. The intention of vegetable farmers in China to adopt the Internet of Things (IoT) is studied.

2. The UTAUT and TOE theories are integrated to analyze personal, technological, organizational, and environmental factors.

3. Structural equation modeling is used to identify the factors influencing the intention to adopt.

4. The indirect effects of TOE variables on UTAUT variables are also explored.
